# Genetic loci of *Mycoplasma agalactiae* involved in systemic spreading during experimental intramammary infection of sheep

**DOI:** 10.1186/s13567-016-0387-0

**Published:** 2016-10-20

**Authors:** Shivanand Hegde, Martina Zimmermann, Martina Flöck, Rene Brunthaler, Joachim Spergser, Renate Rosengarten, Rohini Chopra-Dewasthaly

**Affiliations:** 1 Institute of Microbiology, Department of Pathobiology, University of Veterinary Medicine Vienna, Veterinaerplatz 1, 1210 Vienna, Austria; 2 Clinic for Ruminants, Department for Farm Animals and Veterinary Public Health, University of Veterinary Medicine Vienna, Veterinaerplatz 1, 1210 Vienna, Austria; 3 Institute of Pathology and Forensic Veterinary Medicine, Department of Pathobiology, University of Veterinary Medicine Vienna, Veterinaerplatz 1, 1210 Vienna, Austria; 4 Department of Pathology, University of Texas Medical Branch, Galveston, TX 77555 USA

## Abstract

Mycoplasmas are amongst the most successful pathogens of both humans and animals yet the molecular basis of mycoplasma pathogenesis is poorly understood. This is partly due to the lack of classical virulence factors and little similarity to common bacterial pathogenic determinants. Using *Mycoplasma agalactiae* as a model we initiated research in this direction by screening a transposon mutant library in the natural sheep host using a negative selection method. Having successfully identified putative factors involved in the colonization of local infection and lymphogenic sites, the current study assessed mutants unable to spread systemically in sheep after experimental intramammary infection. Analysis of distant body sites for complete absence of mutants via SSM PCR revealed that additional set of genes, such as *pdhB*, *oppC*, *oppB*, *gtsB*, MAG1890, MAG5520 and MAG3650 are required for systemic spreading apart from those that were necessary for initial colonization. Additional in vitro studies with the mutants absent at these systemic sites confirmed the potential role of some of the respective gene products concerning their interaction with host cells. Mutants of *pdhB*, *oppC* and MAG4460 exhibited significantly slower growth in the presence of HeLa cells in MEM medium. This first attempt to identify genes exclusively required for systemic spreading provides a basis for further in-depth research to understand the exact mechanism of chronicity and persistence of *M. agalactiae*.

**Electronic supplementary material:**

The online version of this article (doi:10.1186/s13567-016-0387-0) contains supplementary material, which is available to authorized users.

## Introduction

*Mycoplasma agalactiae* is mainly responsible for the contagious agalactia (CA) syndrome in small ruminants. As an economically important disease, CA is enlisted as a notifiable disease by the World Organization for Animal Health. Shedding of the pathogen by diseased or asymptomatic carriers continues for months or years after the initial infection, with a potential risk of transmitting the agent to other susceptible animals [1–3]. Antibiotic therapy tends to reduce the clinical signs, but promotes the carrier state [4]. Hence, chronicity and persistence are hallmarks of *M. agalactiae* infections that make it difficult to eradicate. Due to its well-known clinical signs and the capacity to establish experimental infections, *M. agalactiae* serves as a useful model to study mycoplasma pathogenesis in its natural host.

Genome sequencing of the *M. agalactiae* PG2 type strain revealed that several of its genetic loci have been horizontally transferred from the mycoides cluster [5]. However, its virulence mechanisms and factors involved in pathogenesis and disease progression are yet to be understood at the molecular level. Our effort to decipher the pathogenesis of *M. agalactiae* was initiated by gene disruption through homologous recombination and transposon mutagenesis [6, 7]. These studies have demonstrated the role of the *vpma* locus in *M. agalactiae* pathogenesis [8]. Also, recently we attempted to identify potential pathogenicity factors of *M. agalactiae* in the natural sheep host by negative selection of transposon mutants [9]. Other independent studies using transposon mutants were able to show the role of different genomic loci in interaction with host cells [10] and the results obtained were further evaluated in vivo to conclude that the NIF locus was important for the pathogenicity of *M. agalactiae* [11]. Recently, we have shown that *M. agalactiae* can invade eukaryotic cells in vivo and in vitro and also demonstrated its ability to disseminate systemically throughout the body of the infected host after experimental intramammary infection [12]. However, a comprehensive analysis of the molecular genetic factors involved in the systemic spread of *M. agalactiae* in its natural host has not been performed.

Systemic spread of *M. agalactiae* after intramammary infection is well established [12]. In a recent experimental infection trial using pools of transposon mutants for intramammary infection of lactating ewes, we identified transposon mutants absent at the local site of infection and lymph nodes (LNs) [9]. The present study is an extension of the latter work, where we have further assessed the presence or absence of specific mutants in several inner organs distant from the site of inoculation. These data reveal the importance of additional genes required for systemic spread inside the host. The identified mutants belonging to different functional categories were analyzed in vitro using cell cultures to establish their role in interactions with host cells.

## Materials and methods

### Mycoplasma cultures and growth conditions

The *M. agalactiae* type strain PG2 [5, 13] and all mycoplasma cultures were grown as described previously [9]. Gentamicin sulphate (50 µg/mL) was added when propagating transposon mutants.

### Transposon mutants and identification of insertion sites

Transposon Tn*4001*mod [14] was used to construct *M. agalactiae* insertional mutants, as described previously [7]. Transposon insertion sites were identified using a recently described novel sequencing method [9] based on ligation-mediated QTD PCR [15]. The position of the transposon in the genome was determined by nucleotide BLAST in Molligen 3.0 [16].

### Growth analysis and stability of transposon mutants

Growth rate and insertion stability of each of the transposon mutants was assessed in Aluotto medium as described previously [9].

### Signature sequence mutagenesis (SSM) PCR

SSM PCR is based on genetic footprinting for the identification of attenuated mutants in vivo [17] and was modified and performed on *M. agalactiae* as described previously [9].

### Animals and ethical statement

Screening was performed in 1.5 to 2 year-old lactating ewes of a local breed attested to be free of mycoplasmas and other pathogenic bacteria. Experiments were conducted according to the guidelines of the Ethics Committee of the University of Veterinary Medicine Vienna and the Austrian Federal Ministry for Science and Research (approval numbers BMWF-68.205/002-II/3b/2011 and BMWF-68.205/0104-II/3b/2012).

### Initial and secondary screening of transposon mutants

A total of 9 sheep were used in the initial round of screening to test 45 different transposon mutants as described previously [9] except that some additional distant organs, including the spleen, liver, lungs, uterus, kidneys and synovial fluids from stifle joints were also analyzed. Five mutants were kept common between the groups as “watermarks”, and are underlined in Table 1.

**Table 1:** **List of mutants screened in the initial round of screening in an intramammary sheep infection model**

Group I-15 mutants		Group II-17 mutants		Group III-19 mutants	
Mutant	Genes disrupted	Mutant	Genes disrupted	Mutant	Genes disrupted
*Apo7*	*Hypothetical Protein (MAG1890)*	*Apo2-12*	*Hypothetical protein, predicted lipoprotein (MAG1050)*	3-20	NADH oxidase (*nox*)
Apo6	Sugar isomerase (*araD*)	1-3-0	Hypothetical protein (MAG2810)	13	VpmaX
26	Oligopeptide transporter ATP binding protein (*oppF*)	15-2	Hypothetical protein, predicted lipoprotein (MAG6200)	14	DNA methylase (MAG1790)
3-15	Elongation factor G (*fusA*)	170	Hypothetical protein (MAG0110)	*137*	*Hypothetical protein (MAG4460)*
*6-9-2*	*Oligopeptide ABC transporter permease (oppC)*	*3-29*	*Hypothetical protein/Vpma like predicted lipoprotein (MAG2540)*	8-1	Deoxyguaosine kinase (*dgk*)
*6-14*	*Hexose P transport protein (uhpT)*	4-1-0	GTP-dependent nucleic acid-binding protein (engD)	1-2-0	Pentitol posphotransferase enzyme II (MAG6360)
6-21	Cell division protein (*mraZ*)	8 + 1-4	ABC transporter ATP-binding protein (MAG5960)	96-2	ATP synthase (*atpA*)
*6-29*	*Pyruvate dehydrogenase beta subunit (pdhB)*	8 + 1-8	Transport protein SGAT (*ulaA*)	5-1	Hypothetical protein (MAG0250)
*6-32*	*Phosphate acetyl transferase (eutD)*	*3-4-0*	*Hypothetical protein (MAG3390)*	6-13	Hypothetical protein (MAG1570)
7 + 1-8	Hypothetical protein (MAG4010)	9-28	Conserved hypothetical protein (MAG0390)	*7* *+* *1-3*	*Conserved hypothetical protein (MAG3650)*
*9-40*	*Glycerol ABC transporter permease component (gtsB)*	62	Lipoate Protein Ligase A (*lplA*)	*23*	*Aminopeptidase (MAG5520)*
3-1	Phosphoenol- pyruvate phosphotransferase ( *ptsL* )	3-1	Phosphoenolpyruvate Phosphotransferase ( *ptsL* )	3-1	Phosphoenolpyruvate Phosphotransferase ( *ptsL* )
8 + 1-5	Ribonuclease H II ( *rnhB* )	8 + 1-5	Ribonuclease H II ( *rnhB* )		
*9-31*	*Alcohol dehydrogenase (adhT) and NADH oxidase (nox)*			*9-31*	*Alcohol dehydrogenase (adhT) and NADH oxidase (nox)*
6-15	Esterase/lipase ( *lip* )			6-15	Esterase/lipase ( *lip* )
		*81-1*	*Oligopeptide ABC transporter permease (oppB)*	*81-1*	*Oligopeptide ABC transporter permease (oppB)*
		9-8	Dihydrolipoamide dehydrogenase (*pdhD*)	6-12	Transcriptional regulator (MAG6310)
		212	Type III restriction modification system (MAG1530)	San5	Predicted lipoprotein (MAG3120)
		9-16	Conserved hypothetical protein (MAG1450)	3-23	Lipoprotein (MAG6410)
				6-15	VpmaY

Mutants kept common (“watermarks”) between the groups are underlined and those shortlisted for secondary screening showing >95% absence are in italics.

Based on the SSM PCR results and a cut-off for absence of ≥95%, a total of 14 mutants were found to be absent in udders, LNs and systemic sites during initial screening. These mutants were re-screened in a second round of confirmatory screening in three sheep that were also infected via the intramammary route using the same procedures as described previously [9]. Apart from all the tissue samples tested during the initial screening experiment, the brain, heart, and carpal joint tissues were also examined. Furthermore, the presence or absence of *M. agalactiae* in all the tissue samples from the mutant-infected animals was compared with the systemic spread of wild type *M. agalactiae* strain PG2, which was experimentally inoculated into two sheep in parallel as a positive control group [12].

### Cell culture

HeLa-229 (ATCC CCL-2.1) and J774A.1 (ATCC TIB-67) cell lines certified to be free of mycoplasmas were purchased from the American Type Culture Collection (ATCC; Manassas, USA). HeLa-229 cells were maintained in minimum essential medium (MEM) with 10% heat-inactivated foetal bovine serum (FBS) and J774A.1 cells were propagated in DMEM with 10% FBS and 2 mM l-glutamine (Sigma). Trypsin and 1× PBS were purchased from PAA Laboratories GmbH (Pasching, Austria) or Sigma-Aldrich. All cell cultures were regularly checked for mycoplasma contamination by culture and PCR techniques.

### Quantitative growth analysis in presence of mammalian cells

The ability of the mutants and the wild type PG2 strain to grow in the presence of HeLa cells was examined quantitatively by individual co-cultivation experiments. The mutants and wild type strain PG2 were grown as described before. PG2 and mutant cultures were diluted in MEM and inoculated onto 4 × 10^4^ HeLa cells/well (MOI of 10–50) in 24-well tissue culture plates (CELLSTAR® Greiner Bio-One GmbH, Germany). The infected cells were incubated at 37 °C with 5% CO_2_ for 48 h. HeLa cells were trypsinized and cell suspensions were serially diluted in SP4 medium before plating on SP4 agar plates containing appropriate antibiotics. Control wells without HeLa cells did not yield any PG2 or mutant strains’ growth in MEM. All the experiments were carried out at least three times as duplicates. The results are represented as doubling times calculated using the following formula:

Doubling time = (t2 − t1) × (log (2)/log (cfu at t2/cfu at t1)

t1 = Starting time of assay; t2 = End time of assay

### Macrophage cytotoxicity assay

The ability of *M. agalactiae* to kill infected J774.A1 murine macrophages was assessed quantitatively using a colorimetric assay. The release of lactate dehydrogenase (LDH) was quantified using the CytoTox 96® Non-Radioactive cytotoxicity assay kit (Promega). Initially, pilot experiments with uninfected J774A.1 cells were performed to determine the optimum number of target cells to be used with the CytoTox 96® assay according to the manufacturer’s instructions. A 96-well V-bottom microtitre plate containing 5 × 10^3^ J774.A1 cells/well was inoculated separately with *M. agalactiae* wild type strain PG2 and each of the 14 different transposon mutants to get an MOI of about 100. Uninfected J774.A1 cell controls were observed to be intact. The plates were centrifuged at 250 × *g* for 4 min at room temperature and incubated at 37 °C with 5% CO_2_ for 24 h. The released LDH, an indicator of macrophage cell lysis, was calculated using the formula provided with the kit protocol and expressed as a percentage of cytotoxicity. The assay was performed in triplicate at least twice.

### Statistical analysis

The SSM PCR results for each mutant were expressed as average percentage absence ± standard deviation from each group of three sheep. Cut-off criteria of ≥95% and 100% absence was used for considering the mutants absent during the initial and secondary rounds of screening, respectively, and were compared using the Mann–Whitney test [18]. The results of the in vitro assays were compared using the unpaired parametric Student’s *t* test using GraphPad Prism 5 (Graphpad Software Inc, CA, USA), and a value of *p* < 0.05 was considered significant.

## Results

### Identification of *M. agalactiae* genes required for systemic spread

A total of 45 mutants, which exhibited stable transposon insertions and normal growth profiles except *pdhB* mutant [9, 19], were tested in the initial round of screening by intramammary infection of lactating ewes in three different groups, as described in Table 1. Two weeks post-infection (pi), samples of the right and left kidneys, lungs, uterus, liver, spleen and synovial fluids from stifle joints were checked for the presence of *M. agalactiae* by culture and PCR [20], and if positive then for the presence or absence of specific mutants via SSM PCR. Liver, spleen and left lungs were found to be negative for the presence of *M. agalactiae* in all the nine sheep, whereas the uterus, right lungs, kidneys and synovial fluids from stifle joints were positive in some sheep. An additional table summarizes the frequency of isolation of *M. agalactiae* from various systemic sites tested in this study (see Additional file 1).

SSM PCR analysis of *M. agalactiae* positive samples revealed the presence or absence of specific mutants in each of the three sheep of each infected group. Several mutants with disruptions in genes including *rnhII*, *ptsL*, *fusA*, *araD* and *lplA* were found at systemic sites including the uterus and synovial fluids of the stifle joints, thereby demonstrating that these gene defects do not affect their ability to colonize these inner organs. However, a total of 14 mutants, including the “watermark” mutants 9-31 and 81-1, had an average absence percentage of ≥95% (Table 1) in all the tested samples.

As it was difficult to perform individual challenge experiments in sheep with all the 14 attenuated mutants for reasons described previously [9], a second round of confirmatory screening was carried out, as is usually done during negative selection studies [21, 22]. Having confirmed the stability of the transposon, the 14 attenuated mutants were also shown to have no competitive growth deficits when subjected to an in vitro mixed culture growth assay [9]. Analyses of tissue samples from heart, brain and carpal joint (left and right) in addition to lungs, kidneys, spleen, liver, uterus and synovial fluids of the stifle joints were performed to detect the presence of mycoplasmas in systemic sites. *M. agalactiae* wild type strain PG2-infected sheep served as parallel positive controls [12]. Compared to the latter group, in which several systemic sites were positive for *M. agalactiae* (Additional file 1), none of the three sheep infected with the 14 transposon mutants had detectable *M. agalactiae* in any of the 13 systemic sites tested per sheep by PCR and culture, using both direct plating and enrichment studies. Seven of these mutants were exclusively absent only at the systemic sites and were present in the infected udders and surrounding lymph nodes (Table 2). The 100% absence of these 14 mutants in all three sheep of the confirmatory screening clearly demonstrates their inability to disseminate into distant body sites. The wild type PG2 strain, on the other hand, was able to cause systemic infection, as confirmed by PCR (Additional file 1) and immunohistochemical analysis [12]. Hence, these results indicate that the transposon insertions into specific genes were responsible for the inability of these mutants to spread systemically and colonize distant body sites.

**Table 2:** Mutants showing complete absence exclusively at the systemic sites of all three infected sheep

Mutant	Gene	Pathogenicity associated studies
*Apo7*	MAG1890	45
*6-9-2*	MAG0360_oppC	29-34
*81-1*	MAG0370_oppB	
*6-29*	MAG0940_pdhB	19, 40-44
*9-40*	MAG2320_gtsB	28, 36-37
*7* *+* *1-3*	MAG3650	
*23*	MAG5520_aminopeptidase	44

In order to estimate the reproducibility of results obtained with common “watermark” mutants between different groups, the Mann–Whitney test [18] was applied. No significant difference was detected in average percentage of absence (*p* > 0.05) of attenuated mutants that are common between the groups.

### Phenotypic analysis of attenuated mutants

As the in vivo screening identified a relatively high number of genes required for systemic spread, and especially because we could not test each of the 14 mutants in individual infection trials to compare with wild type PG2 strain, we decided to assess their in vitro interactions with host cells in cell culture. The ability of the mutants to grow in the presence of mammalian cells like HeLa and their involvement in the killing of murine macrophages have been previously reported to be important characteristics for evaluating their potential role in pathogenicity and systemic spread [10, 11, 23, 24]. Moreover, as we had earlier reported the presence of *M. agalactiae* wild type strain PG2 in lung macrophages of infected sheep [12], we wanted to check if there was any defect in the ability of the mutants to infect non-phagocytic cells and if they caused any cytotoxicity in macrophages.

**Figure 1:**
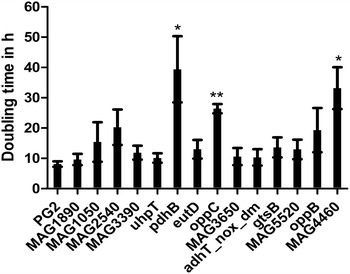
**Growth analysis of selected transposon mutants during co-cultivation with HeLa cells.** Doubling time, in presence of HeLa cells 48 h pi, of the wild type strain PG2 and transposon mutants deficient in systemic spreading is shown. Each bar represents the average doubling time ± standard deviation of three independent experiments done in duplicates. *p < 0.05; **p < 0.005.

HeLa cells were co-cultivated with the wild type strain PG2 and also separately with each of the 14 mutants that could not spread systemically and were not isolated from any of the distant inner organs of infected sheep. The in vitro results showed that *pdhB*, *oppC* and MAG4460 mutants exhibited significantly slower growth in the presence of HeLa cells as compared to the PG2 strain (Figure 1).

**Figure 2:**
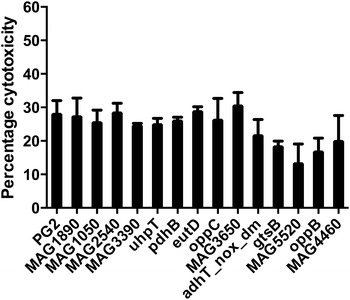
**Macrophage cytotoxicity assay of selected transposon mutants in murine macrophages.** Percentage cytotoxicity of the wild type strain PG2 and selected transposon mutants in J774.A1 cells at 48 h pi is depicted. Each bar represents the average percentage cytotoxicity ± standard deviation of three independent experiments done in triplicates.

The ability of the mutants to kill murine macrophages was assessed in comparison to the wild type strain using a non-radioactive assay. Although none of the mutants had significant alterations in their killing capacity compared to the wild type PG2 strain, the mutant MAG5520 was nearly two-fold deficient (*p* > 0.05) in its cytotoxic effect on macrophages (Figure 2). This mutant showed only 13% macrophage killing at 24 h pi, whereas the wild type strain exhibited a cytotoxic rate of about 27%. All the other 14 mutants demonstrated a cytotoxic potential comparable to that of the wild type strain (Figure 2).

## Discussion

**Figure 3:**
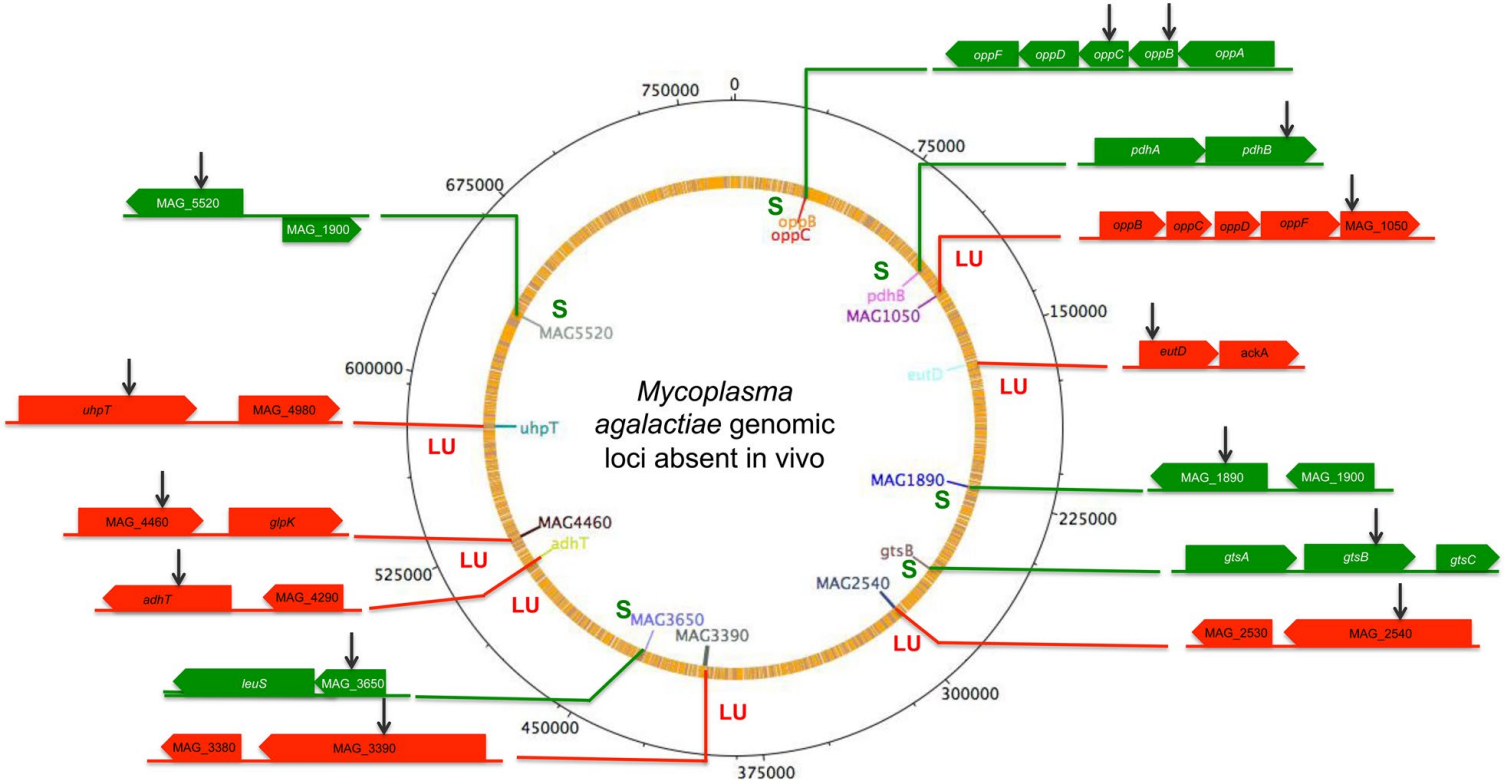
**Genomic loci that play a potential role in the systemic spread of** ***M. agalactiae***. Transposon mutants completely absent in LNs and udders are depicted in red (LU) whereas those that were present at latter sites but exclusively absent only at systemic sites are shown in green (S). The black arrows represent the location of transposon insertion sites in the respective genes.

Bacteria belonging to the genus *Mycoplasma* are capable of causing chronic and persistent infections in a variety of hosts. As a pathogen of small ruminants, *M. agalactiae* is capable of subverting the host immune response likely through its sophisticated Vpma antigenic variation system [8], and by intracellular residence and spread to distant safer host niches [12]. Until our recent study [9], which identified mutants unable to colonize the initial local infection site and associated LNs during experimental intramammary infection, the molecular basis of *M. agalactiae* pathogenesis had been little studied. The aim of the study presented here was to identify in the sheep intramammary infection model genetic factors of *M. agalactiae* that play a role in its systemic spreading to distant sites in the natural host. Our previous analysis of the local infection site revealed the absence of 7 mutants belonging to different functional categories [9]. Further analysis of distant inner organs and host sites has now revealed 7 additional transposon mutants that exclusively show 100% absence in all three sheep at these sites (Table 2). The genomic loci corresponding to these mutants seem to play an exclusive and important role in the systemic spreading of *M. agalactiae* from the site of initial infection (Figure 3). Unlike the other seven mutants that were unable to colonize the local infection site (udder) and LNs [9], the additional set of 7 mutants were absent only at systemic sites (Table 2). These results suggest that having established a successful infection at the local sites, such as udders, this ruminant pathogen employs additional factors to spread systemically to newer distant host niches. Hence, the mutants absent at the distant inner organs not only correspond to genes required for initial infection but also those that play important roles in the systemic spreading of the pathogen (Figure 3). Among the mutants absent exclusively at the distant systemic sites, some of the corresponding genes, such as *oppB*, *oppC* and *gtsB*, are reported to be acquired from mycoides cluster through horizontal gene transfer [5], perhaps under selection pressure to possibly provide additional advantages of systemic spreading and thus persistence. Hence, genetic factors identified for *M. agalactiae* pathogenicity could also be important in the infection biology of the mycoplasma species belonging to the mycoides cluster.

Metabolic proteins and transporters are known to play a crucial role in mycoplasma pathogenesis [25–28]. Our study identified two genes coding for the permease component of an oligopeptide transporter, namely *oppB* and *oppC*. Although *Mycoplasma hominis* OppA has been implicated in mammalian cell adhesion and apoptotic cell death [29, 30], involvement of permease components in virulence has been so far reported only in pathogenic bacteria other than mycoplasmas [31–34]. In addition, the mutant *oppC* is also growth-deficient in the presence of HeLa cells, indicating that the transport of peptides is important for both in vitro and in vivo survival of *M. agalactiae* in presence of host cells. Based on these in vitro and in vivo data, nutrient acquisition such as the uptake of small peptides seems to play an important role in the survival of *M. agalactiae* in the host. Additionally, like several other mycoplasma species, *M. agalactiae* has retained both the parent Opp operon as well as the horizontally transferred locus [35], further indicating its significant role in pathogenesis. Likewise, glycerol seems to play a crucial role in mycoplasma pathogenesis as a substrate for toxic metabolic products like H_2_O_2_ [28, 36]. In this study, a mutant defective in the permease component of a glycerol ABC transporter (*gtsB*) was unable to spread to or survive in the systemic sites. The genes involved in transport and metabolism of glycerol have been previously reported as important virulence factors [25–27, 36]. The glycerol transport locus is well characterised in members of the mycoides cluster [37] and has been shown to play a role in virulence [28]. As *M. agalactiae* has probably acquired its *gtsABC* from the mycoides cluster through horizontal gene transfer [35], it is likely that this locus also plays a role in its pathogenesis. However, unlike the members of the mycoides cluster, *M. agalactiae* lacks the gene for the active transport of glycerol, as well as that encoding for glycerol phosphate oxidase, both of which are primarily responsible for H_2_O_2_ production. Taking into account that the *gtsB* mutant was found to be attenuated in this study, further analyses could lead to interesting findings.

Apart from oligopeptide transporter genes, a mutant with a disrupted *pdhB* gene, coding for the beta subunit of the pyruvate dehydrogenase complex, was also absent in the systemic sites. This gene is responsible for the metabolism of pyruvate, which is the major energy yielding process in *M. agalactiae* [38]. The *pdhB* mutant showed reduced growth in axenic medium during logarithmic phase, but was recovered in vivo from the udder and LNs of infected animals in the confirmatory screening experiment [9, 19], indicating its relative survival in local sites of infection. Additionally, this mutant also showed reduced invasion into HeLa cells [19], indicating its role in host cell invasion and the significance of the latter in the systemic spread of *M. agalactiae*. In other words, the absence of this mutant at distant systemic sites shows its inability to disseminate throughout the body to cause systemic infection, which in turn could be due to its reduced invasiveness. As systemic spread and cell invasion are often related [12, 39], it is possible that the mutant’s inability to cross the epithelial barrier might be responsible for its deficient spreading capacity. Additionally, the *pdhB* mutant also showed a growth defect in the presence of HeLa cells in vitro and this might be due to its inability to interact with host cell proteins. Earlier studies with *Mycoplasma pneumoniae* have shown that PdhB is surface localised [40] and binds fibronectin and plasminogen [41, 42]. Thus, like many other mycoplasmas [43, 44], *M. agalactiae* might also be utilising a single gene product to carry out multiple functions.

Aminopeptidases play important roles in pathogenesis of bacteria by providing a source of amino acids from exogenous proteins by destroying host immunological effector peptides. A *M. agalactiae* mutant containing transposon insertion in the gene coding for glutamyl aminopeptidase (MAG5520) was unable to spread inside the infected sheep. This mutant also showed reduced killing of murine macrophages as measured in the cytotoxicity assay. A similar protein in *Mycoplasma hyopneumoniae* was shown to be surface-located, involved in adhesion and capable of binding and cleaving plasminogen [44]. Interestingly, the latter exhibits more than 50% identity to the *M. agalactiae* aminopeptidase and might have the same characteristics. Hence, it is possible that disruption of this gene results in reduced ability to generate amino acids for *M. agalactiae’s* survival inside the host.

In addition to mutants in annotated genes, this study also identified loci coding for hypothetical or conserved hypothetical proteins. Mutants with disruptions in MAG1890 and MAG3650 genes encoding proteins of unknown functions were found attenuated only in systemic sites but were recovered from the udders and LNs. An independent study had earlier shown that transposon insertion in MAG1890 resulted in reduced interaction of *M. agalactiae* with goat embryo fibroblast cells [45]. However, our studies with HeLa cells did not show a significant difference in co-culture growth (*p* > 0.05) and this may be due to the differences in the cell lines used. Sequence analysis of MAG3650 showed 86% identity to a putative membrane protein of *Mycoplasma bovis* (*MBOVPG45_0454*). Since these ruminant mycoplasmas are phylogenetically closely related and even cause similar infections in their respective hosts, they also might share their protein functions. Thus, MAG3650’s corresponding homolog MBOVPG45_0454 might also play a critical role in the pathogenicity of the economically more important *M. bovis*. These results indicate that, apart from known lipoproteins, transporters and metabolic proteins acting as virulence factors, hypothetical proteins might also play a crucial role in *M. agalactiae* pathogenesis.

Altogether, the study presented here has shown the possible role of several genomic loci (Figure 3) involved in systemic spreading of *M. agalactiae* during experimental intramammary infection of lactating ewes. The results provide a better understanding of how *M. agalactiae* utilizes its genomic machinery to establish systemic infection in its natural host. To our knowledge, this is the first report describing the potential role of a set of genetic factors involved in systemic infections of a ruminant mycoplasma. Further studies of these factors in individual infection experiments could pave the way in identifying a suitable mutant that could be developed further as a good vaccine candidate.

## Additional file

**Additional file 1.** **Table summarizing the frequency of isolation of** ***M. agalactiae*** **from various systemic sites of sheep infected with transposon mutant pools**. The additional column compares the results with the presence of wild type PG2 strain in samples procured from parallel infected control sheep [12]. NA: Not applicable; L: Left; R: Right.

## References

[1] Bergonier D, Berthelot X, Poumarat F (1997). Contagious agalactia of small ruminants: current knowledge concerning epidemiology, diagnosis and control. Rev Sci Tech.

[2] Bergonier D, Poumarat F (1996). Contagious agalactia of small ruminants: epidemiology, diagnosis and control. Rev Sci Tech.

[3] Corrales JC, Esnal A, De la Fe C, Sánchez A, Assunçao P, Poveda JB, Contreras A (2007). Contagious agalactia in small ruminants. Small Rumin Res.

[4] Nicholas RA (2002). Improvements in the diagnosis and control of diseases of small ruminants caused by mycoplasmas. Small Rumin Res.

[5] Sirand-Pugnet P, Lartigue C, Marenda M, Jacob D, Barre A, Barbe V, Schenowitz C, Mangenot S, Couloux A, Segurens B, de Daruvar A, Blanchard A, Citti C (2007). Being pathogenic, plastic, and sexual while living with a nearly minimal bacterial genome. PLoS Genet.

[6] Chopra-Dewasthaly R, Citti C, Glew MD, Zimmermann M, Rosengarten R, Jechlinger W (2008). Phase-locked mutants of *Mycoplasma agalactiae*: defining the molecular switch of high-frequency Vpma antigenic variation. Mol Microbiol.

[7] Chopra-Dewasthaly R, Zimmermann M, Rosengarten R, Citti C (2005). First steps towards the genetic manipulation of *Mycoplasma agalactiae* and *Mycoplasma bovis* using the transposon Tn*4001*mod. Int J Med Microbiol.

[8] Chopra-Dewasthaly R, Baumgartner M, Gamper E, Innerebner C, Zimmermann M, Schilcher F, Tichy A, Winter P, Jechlinger W, Rosengarten R, Spergser J (2012). Role of Vpma phase variation in *Mycoplasma agalactiae* pathogenesis. FEMS Immunol Med Microbiol.

[9] Hegde S, Hegde S, Zimmermann M, Flock M, Spergser J, Rosengarten R, Chopra-Dewasthaly R (2015). Simultaneous identification of potential pathogenicity factors of *Mycoplasma agalactiae* in the natural ovine host by negative selection. Infect Immun.

[10] Baranowski E, Guiral S, Sagne E, Skapski A, Citti C (2010). Critical role of dispensable genes in *Mycoplasma agalactiae* interaction with mammalian cells. Infect Immun.

[11] Baranowski E, Bergonier D, Sagne E, Hygonenq MC, Ronsin P, Berthelot X, Citti C (2014). Experimental infections with *Mycoplasma agalactiae* identify key factors involved in host-colonization. PLoS One.

[12] Hegde S, Hegde S, Spergser J, Brunthaler R, Rosengarten R, Chopra-Dewasthaly R (2014). *In vitro* and in vivo cell invasion and systemic spreading of *Mycoplasma agalactiae* in the sheep infection model. Int J Med Microbiol.

[13] Solsona M, Lambert M, Poumarat F (1996). Genomic, protein homogeneity and antigenic variability of *Mycoplasma agalactiae*. Vet Microbiol.

[14] Knudtson KL, Minion FC (1993). Construction of Tn*4001* lac derivatives to be used as promoter probe vectors in mycoplasmas. Gene.

[15] Sharma VM, Chopra R, Ghosh I, Ganesan K (2001). Quantitative target display: a method to screen yeast mutants conferring quantitative phenotypes by ‘mutant DNA fingerprints’. Nucleic Acids Res.

[16] Barre A, de Daruvar A, Blanchard A (2004). MolliGen, a database dedicated to the comparative genomics of *Mollicutes*. Nucleic Acids Res.

[17] Hudson P, Gorton TS, Papazisi L, Cecchini K, Frasca S, Geary SJ (2006). Identification of a virulence-associated determinant, dihydrolipoamide dehydrogenase (*lpd*), in *Mycoplasma gallisepticum* through in vivo screening of transposon mutants. Infect Immun.

[18] Mann HB, Whitney DR (1947). On a test of whether one of 2 random variables Is stochastically larger than the other. Ann Math Stat.

[19] Hegde S, Rosengarten R, Chopra-Dewasthaly R (2015). Disruption of the *pdhB* pyruvate dehydrogenase gene Affects colony morphology, in vitro growth and cell invasiveness of *Mycoplasma agalactiae*. PLoS One.

[20] Chavez Gonzalez YR, Ros Bascunana C, Bolske G, Mattsson JG, Fernandez Molina C, Johansson KE (1995). In vitro amplification of the 16S rRNA genes from *Mycoplasma bovis* and *Mycoplasma agalactiae* by PCR. Vet Microbiol.

[21] Flashner Y, Mamroud E, Tidhar A, Ber R, Aftalion M, Gur D, Lazar S, Zvi A, Bino T, Ariel N, Velan B, Shafferman A, Cohen S (2004). Generation of *Yersinia pestis* attenuated strains by signature-tagged mutagenesis in search of novel vaccine candidates. Infect Immun.

[22] Hendrixson DR, DiRita VJ (2004). Identification of *Campylobacter jejuni* genes involved in commensal colonization of the chick gastrointestinal tract. Mol Microbiol.

[23] Allewelt M, Coleman FT, Grout M, Priebe GP, Pier GB (2000). Acquisition of expression of the *Pseudomonas aeruginosa* ExoU cytotoxin leads to increased bacterial virulence in a murine model of acute pneumonia and systemic spread. Infect Immun.

[24] Weiss DS, Brotcke A, Henry T, Margolis JJ, Chan K, Monack DM (2007). *In vivo* negative selection screen identifies genes required for *Francisella* virulence. Proc Natl Acad Sci U S A.

[25] Pilo P, Vilei EM, Peterhans E, Bonvin-Klotz L, Stoffel MH, Dobbelaere D, Frey J (2005). A metabolic enzyme as a primary virulence factor of *Mycoplasma mycoides* subsp. *mycoides* small colony. J Bacteriol.

[26] Grosshennig S, Schmidl SR, Schmeisky G, Busse J, Stulke J (2013). Implication of glycerol and phospholipid transporters in *Mycoplasma pneumoniae* growth and virulence. Infect Immun.

[27] Schmidl SR, Otto A, Lluch-Senar M, Pinol J, Busse J, Becher D, Stulke J (2011). A trigger enzyme in *Mycoplasma pneumoniae*: impact of the glycerophosphodiesterase GlpQ on virulence and gene expression. PLoS Pathog.

[28] Vilei EM, Frey J (2001). Genetic and biochemical characterization of glycerol uptake in *Mycoplasma mycoides* subsp. *mycoides SC*: its impact on H_2_O_2_ production and virulence. Clin Diagn Lab Immunol.

[29] Hopfe M, Dahlmanns T, Henrich B (2011). In *Mycoplasma hominis* the OppA-mediated cytoadhesion depends on its ATPase activity. BMC Microbiol.

[30] Hopfe M, Henrich B (2008). OppA, the ecto-ATPase of *Mycoplasma hominis* induces ATP release and cell death in HeLa cells. BMC Microbiol.

[31] Flores-Valdez MA, Morris RP, Laval F, Daffe M, Schoolnik GK (2009). *Mycobacterium tuberculosis* modulates its cell surface via an oligopeptide permease (Opp) transport system. FASEB J.

[32] Kerr AR, Adrian PV, Estevao S, de Groot R, Alloing G, Claverys JP, Mitchell TJ, Hermans PW (2004). The Ami-AliA/AliB permease of *Streptococcus pneumoniae* is involved in nasopharyngeal colonization but not in invasive disease. Infect Immun.

[33] Wang CH, Lin CY, Luo YH, Tsai PJ, Lin YS, Lin MT, Chuang WJ, Liu CC, Wu JJ (2005). Effects of oligopeptide permease in group a streptococcal infection. Infect Immun.

[34] Wu TK, Wang YK, Chen YC, Feng JM, Liu YH, Wang TY (2007). Identification of a *Vibrio furnissii* oligopeptide permease and characterization of its in vitro hemolytic activity. J Bacteriol.

[35] Sirand-Pugnet P, Citti C, Barre A, Blanchard A (2007). Evolution of *Mollicutes*: down a bumpy road with twists and turns. Res Microbiol.

[36] Hames C, Halbedel S, Hoppert M, Frey J, Stulke J (2009). Glycerol metabolism is important for cytotoxicity of *Mycoplasma pneumoniae*. J Bacteriol.

[37] Djordjevic SP, Vilei EM, Frey J (2003). Characterization of a chromosomal region of *Mycoplasma* sp. bovine group 7 strain PG50 encoding a glycerol transport locus (*gtsABC*). Microbiology.

[38] Miles RJ, Wadher BJ, Henderson CL, Mohan K (2008). Increased growth yields of *Mycoplasma* spp. in the presence of pyruvate. Lett Appl Microbiol.

[39] Much P, Winner F, Stipkovits L, Rosengarten R, Citti C (2002). *Mycoplasma gallisepticum*: influence of cell invasiveness on the outcome of experimental infection in chickens. FEMS Immunol Med Microbiol.

[40] Grundel A, Friedrich K, Pfeiffer M, Jacobs E, Dumke R (2015). Subunits of the pyruvate dehydrogenase cluster of *Mycoplasma pneumoniae* are surface-displayed proteins that bind and activate human plasminogen. PLoS One.

[41] Thomas C, Jacobs E, Dumke R (2013). Characterization of pyruvate dehydrogenase subunit B and enolase as plasminogen-binding proteins in *Mycoplasma pneumoniae*. Microbiology.

[42] Dallo SF, Kannan TR, Blaylock MW, Baseman JB (2002). Elongation factor Tu and E1 beta subunit of pyruvate dehydrogenase complex act as fibronectin binding proteins in *Mycoplasma pneumoniae*. Mol Microbiol.

[43] Dumke R, Hausner M, Jacobs E (2011). Role of *Mycoplasma pneumoniae* glyceraldehyde-3-phosphate dehydrogenase (GAPDH) in mediating interactions with the human extracellular matrix. Microbiology.

[44] Robinson MW, Buchtmann KA, Jenkins C, Tacchi JL, Raymond BB, To J, Roy Chowdhury P, Woolley LK, Labbate M, Turnbull L, Whitchurch CB, Padula MP, Djordjevic SP (2013). MHJ_0125 is an M42 glutamyl aminopeptidase that moonlights as a multifunctional adhesin on the surface of *Mycoplasma hyopneumoniae*. Open Biol.

[45] Skapski A, Hygonenq MC, Sagne E, Guiral S, Citti C, Baranowski E (2011). Genome-scale analysis of M*ycoplasma agalactiae* loci involved in interaction with host cells. PLoS One.

