# Supplementary material for: Genetic loci of Mycoplasma agalactiae involved in systemic spreading during experimental intramammary infection of sheep
Source: Vet Res. 2016 Oct 20;47:106. doi: 10.1186/s13567-016-0387-0 (PMC5073455; doi:10.1186/s13567-016-0387-0)
Supplement: Supplementary file 1 — Additional file 1. Table summarizing the frequency of isolation of M. agalactiae from various systemic sites of sheep infected with transposon mutant pools. The additional column compares the results with the presence of wild type PG2 strain in samples procured from parallel infected control sheep [12]. NA: Not applicable; L: Left; R: Right. [file 13567_2016_387_MOESM1_ESM.docx]

|  | **Positive samples/total number of samples examined** | | **PG2** [12] |
| --- | --- | --- | --- |
| **Organs/tissue samples** | **Initial screening** | **Secondary screening** |  |
| Liver | 0/9 | 0/3 | + |
| Spleen | 0/9 | 0/3 | - |
| Kidney L | 1/9 | 0/3 | NA |
| Kidney R | 2/9 | 0/3 | + |
| Lung L | 0/9 | 0/3 | + |
| Lung R | 1/9 | 0/3 | + |
| Uterus | 4/9 | 0/3 | + |
| Stifle Joint/ Synovial Fluid L | 3/9 | 0/3 | NA |
| Stifle Joint/  Synovial Fluid R | 7/9 | 0/3 | + |
| Heart | NA | 0/3 | + |
| Brain | NA | 0/3 | + |
| Carpal Joint Capsule L | NA | 0/3 | + |
| Carpal Joint  Capsule R | NA | 0/3 | + |
